# Clinical Predictors of Weaning Failure and Mortality in Individuals With COVID‐19 Undergoing Invasive Mechanical Ventilation: A Retrospective Cohort Study

**DOI:** 10.1155/pm/9316439

**Published:** 2026-04-15

**Authors:** Natália Trindade, Larissa Araújo de Castro, Walter Sepúlveda-Loyola, Carrie Chueiri Ramos Galvan, Josiane Marques Felcar, Ercy Mara Cipulo Ramos, Vanessa Suziane Probst

**Affiliations:** ^1^ Graduate Program in Rehabilitation Sciences, State University of Londrina (UEL) and University of North Paraná (UNOPAR), Londrina, Brazil; ^2^ University Hospital, State University of Londrina (UEL), Londrina, Brazil, uel.br; ^3^ Faculty of Health and Social Sciences, Universidade de Las Americas (UDLA), Santiago, Chile; ^4^ Department of Physiotherapy, State University of Londrina (UEL), Londrina, Brazil, uel.br; ^5^ Research and Graduate Center in Health Sciences (CEPPOS), Health Sciences Center (CCS), State University of Londrina (UEL), Londrina, Brazil, uel.br; ^6^ Department of Physiotherapy, São Paulo State University (UNESP), São Paulo, Brazil, unesp.br

**Keywords:** artificial respiration, COVID-19, intensive care unit, intubation, university hospitals, weaning

## Abstract

**Background:**

During the COVID‐19 pandemic, a significant number of affected individuals experienced acute respiratory failure requiring invasive mechanical ventilation (IMV). Once IMV becomes necessary, substantial efforts are made to conduct a successful ventilatory weaning process and prevent deleterious effects associated with prolonged positive pressure use. Identifying the clinical characteristics of COVID‐19 patients who experienced failure in ventilatory weaning can provide insights to optimize ventilatory management.

**Aim:**

This study is aimed at identifying predictors of weaning failure (WF) from IMV in patients with COVID‐19 and their association with mortality.

**Methods:**

This retrospective cohort study analyzed data obtained from medical records, including personal and clinical information, IMV parameters, ventilatory mechanics, IMV duration, hospital and intensive care unit (ICU) length of stay, and discharge or death date. All COVID‐19 patients admitted between March 2020 and July 2022, aged ≥ 18 years, and requiring IMV were included. Patients were categorized into three groups: weaning success (WS), WF, and no criteria for weaning (NCW). Success was defined as orotracheal prosthesis removal without reintubation within 48 h or, for tracheostomized patients, disconnection from mechanical ventilation for ≥ 48 h.

**Results:**

A total of 2.198 individuals were included, of whom 528 (age 52 [39–61] years, 195 men, and body mass index 29 [26–34] kg/m^2^) met the criteria for weaning and were analyzed (WS: *n* = 380, 195 men; WF: *n* = 148, 89 men). Individuals in the WS group were younger (WS: 52 [39–61] vs. WF: 56 [47–67] years; *p* < 0.0001) and had shorter ICU stay (WS: 16 [10–27] vs. WF: 29 [14–45] days; *p* < 0.0001) and IMV duration (WS: 12 [7–20] vs. WF: 25 [14–37] days; *p* < 0.0001). Independent predictors of WF included age (OR [95% CI]: 1.028 [1.005–1.052]), length of hospital stay (OR [95% CI]: 0.963 [0.937–0.990]), time on IMV (OR [95% CI]: 1.103 [1.059–1.149]), and driving pressure (OR [95% CI]: 3.750 [1.344–10.466]), regardless of gender and comorbidities. Mortality was higher in the WF group (WF: 69% vs. WS: 24%; *p* < 0.0001).

**Conclusion:**

Advanced age, the length of hospitalization, prolonged IMV duration, and compromised respiratory mechanics were the predictors of WF. Furthermore, individuals who experienced WF presented a higher mortality rate compared with those who successfully weaned.

## 1. Introduction

COVID‐19 is a highly contagious infectious disease caused by the Coronavirus 2 (SARS‐CoV‐2) [1, 2], which emerged as a global challenge, impacting public health, the economy, and social relationships worldwide [3]. The disease presents a range of symptoms, from mild to severe, and its spread quickly reached exponential levels. In March 2020, it was officially designated a pandemic by the World Health Organization (WHO) [1, 3–5].

This virus easily binds to respiratory cells through an interaction between its spike protein and the host cells, triggering an inflammatory response that can progress to acute respiratory distress syndrome (ARDS). Beyond the respiratory system, SARS‐CoV‐2 exhibits a multifaceted impact on organs such as the heart, kidneys, and nervous system [5–7].

The diversity of clinical manifestations of COVID‐19 ranges from mild symptoms to severe cases that evolve into acute respiratory failure (ARF) [2, 8, 9]. In such cases, a cytokine storm and clot formation occur, contributing to vascular complications. When ARDS progresses to ARF, the disease presents greater treatment challenges, often requiring the implementation of noninvasive mechanical ventilation (NIMV) or invasive mechanical ventilation (IMV) as part of the treatment [10–12].

IMV has become a crucial intervention in the treatment of severely affected COVID‐19 patients [13–15], especially those who do not qualify for or respond well to NIMV [13]. It not only assists in oxygenating the blood but also alleviates the workload on the patient′s respiratory system, enabling the body to combat the infection [15, 16].

However, its use is associated with setbacks, such as the potential occurrence of ventilator‐induced lung injuries, highlighting the importance of careful monitoring and personalized adjustment of ventilatory parameters to optimize respiratory support and promote faster and more effective recovery [17, 18].

Another challenge is the ventilatory weaning process, which refers to the removal of the patient from IMV. This process can be classified into simple weaning, when extubation occurs successfully on the first attempt without failure; difficult weaning, when the patient fails the first extubation attempt but succeeds after fewer than three spontaneous breathing trials (SBTs) or within 7 days; and prolonged weaning, which requires three or more SBTs or a period greater than 7 days after the first failure to achieve successful extubation [19, 20].

In COVID‐19, the weaning process is complex, made intricate by the heterogeneity of patients [21, 22]. Careful evaluation of the clinical profile is essential to determine the appropriate timing for initiating the process [23]. Prerequisites include discontinuing sedatives, resolving the underlying causes of IMV, and maintaining hemodynamic stability [24–28].

Successful weaning is achieved when the patient remains in spontaneous ventilation for at least 48 h [27]. A thorough understanding of these processes is imperative, given the complex challenges in managing COVID‐19 patients, who exhibit a high prevalence of extubation failure and postweaning mortality [15, 16, 29].

Given this, the present study is aimed at identifying predictors of weaning failure (WF) from IMV in patients with COVID‐19 and their association with mortality.

## 2. Materials and Methods

### 2.1. Study Design and Setting

This article resulted from a master′s degree thesis [30]. This retrospective cohort study was conducted using data from electronic medical records of COVID‐19 patients admitted to the University Hospital of Londrina, Brazil, between March 2020 and July 2022. The study was approved by the university′s ethics committee (Approval Number: 4.327.528).

### 2.2. Participants

The study included patients aged ≥ 18 years with a confirmed COVID‐19 diagnosis who required IMV. Patients were excluded if they lacked complete clinical data or did not meet the weaning criteria.

### 2.3. Weaning Criteria and Readiness Assessment

Patients were considered to have met weaning criteria when they fulfilled the following institutional readiness parameters, which are consistent with international guidelines [19]: PaO_2_/FiO_2_ ≥ 150 with FiO_2_ ≤ 0.4, PEEP ≤ 8 cmH_2_O, hemodynamic stability, adequate level of consciousness (RASS, −2 to 0), absence of significant respiratory acidosis (pH ≥ 7.32), effective spontaneous respiratory drive, manageable secretions, and no new organ dysfunction in the prior 24 h. Once these criteria were met, the clinical team proceeded with a SBT.

### 2.4. Definitions and Grouping

Weaning success (WS) was defined as extubation or mechanical ventilation disconnection for ≥ 48 h without reintubation. WF was defined as patients requiring reintubation or reconnection within 48 h.

### 2.5. Data Collection

Demographic, clinical, and ventilatory data were extracted, including age, sex, comorbidities, duration of IMV and ICU stay, ventilatory parameters, and outcomes (hospital discharge or death).

### 2.6. Statistical Analysis

Statistical analysis was performed using the Statistical Package for the Social Sciences‐23 (SPSS, United States) and GraphPad Prism. Numerical variables were assessed for normality using the Shapiro–Wilk test. Due to the nonnormal distribution of the variables, they were presented as median (interquartile range). Categorical variables were presented as absolute and relative frequencies. For categorical variable assessment, univariate analysis was performed using Fisher′s exact test. The studied individuals were separated into three groups for comparison: WS, WF, and no criteria for weaning (NCW). These comparisons were made using the Kruskal–Wallis test.

The primary objective was to identify predictors of WF from mechanical ventilation, whereas secondary outcomes included ICU length of stay, total hospital length of stay, readmission, and mortality rates. To identify predictors of WF, we employed linear regression using the forward stepwise approach to analyze the relationship between selected independent and dependent variables. The inclusion criteria for variables were based on statistics such as *p* value, adjusted determination coefficient (*R* ^2^ adjusted), odds ratio (OR), and 95% confidence intervals (CIs). In addition, the Kaplan–Meier survival curves were constructed to compare mortality between subjects from WS and WF, with differences assessed using the log‐rank (Mantel–Cox) test. Survival analysis was stratified by WS and WF status. Statistical significance was set at 5% (*p* < 0.05).

## 3. Results

**Figure 1:**
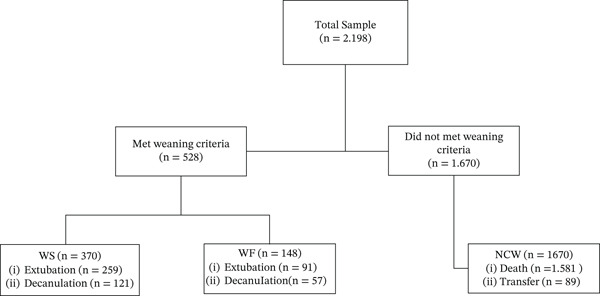
Study flowchart. WS, weaning success; WF, weaning failure; NCW, no criteria for weaning.

A total of 2.198 individuals, as shown in Figure 1, were included, of whom 528 met the criteria for weaning and were analyzed (WS: *n* = 380; 195 men; WF: *n* = 148; 89 men). Individuals in the WS group were younger (WS: 52 [39–61] vs. WF: 56 [47–67] years; *p* < 0.0001), spent less time in the ICU (WS: 16 [10–27] vs. WF: 29 [14–45] days; *p* < 0.0001), and required fewer days on IMV (WS: 12 [7–20] vs. WF: 25 [14–37] days; *p* < 0.0001). A detailed comparison of clinical and ventilatory parameters between groups is presented in Table 1.

**Table 1:** Comparison of characteristics of the total study sample.

Variables	WS (*n* = 380)	WF (*n* = 148)	NCW (*n* = 1670)	*p*
Anthropometric data				
Gender (F/M)	185 (49%)/195 (51%)	59 (40%)/89 (60%)	650 (39%)/1020 (61%)	0.002^a^
Age (years)	52 [39–61]	56 [47–67]	66 [56–75]	< 0.001^a,b,c^
BMI^d^ (kg/m^2^)	29 [26–34]	28 [25–32]	28 [25–33]	0.025^a^
Smoking history				
Never smoked	324 (85%)	121 (82%)	1311 (78%)	0.013^a^
Smoker	15 (4%)	7 (5%)	113 (7%)	
Former smoker	41 (11%)	20 (13%)	245 (15%)	
Symptoms and comorbidities				
No. of symptoms	2.25 (1.6)	2.01 (1.6)	2.06 (1.6)	0.106
No. of comorbidities	1.74 (1.35)	1.84 (1.19)	2.01 (1.35)	< 0.001^a^
Funcionality^e^				
Independent	339 (90%)	121 (83%)	1237 (76%)	< 0.001^a^
Partially dependent	31 (8%)	16 (11%)	242 (15%)	
Dependent	8 (2%)	8 (6%)	137 (9%)	
Chest tomography (% involvement)^f^				
< 25%	19 (6%)	4 (3%)	76 (6%)	0.764
25%–50%	39 (12%)	18 (14%)	194 (14%)	
51%–75%	129 (38%)	42 (34%)	452 (34%)	
> 75%	153 (45%)	61 (49%)	623 (46%)	
Use of NIMV Y/N (%)	163 (43%)/217 (57%)	60 (40%)/88 (60%)	395 (24%)/1275 (81%)	< 0.001^a,c^
IMV				
Ventilation mode^g^				
VCV	334 (88%)	135 (91%)	1521 (94%)	< 0.001^a^
PCV	22 (6%)	5 (3%)	90 (5%)	
PSV	22 (6%)	8 (6%)	15 (1%)	
Tidal volume^h^				
≤ 6 mL/kg	233 (67%)	80 (58%)	665 (43%)	< 0.001^a,c^
6.1–7.9 mL/kg	102 (29%)	48 (34%)	650 (42%)	
> 8 mL/kg	13 (4%)	11 (8%)	241 (15%)	
Respiratory rate^i^				
≤ 25 rpm	37 (10%)	11 (8%)	62 (4%)	< 0.001^a,c^
26–30 rpm	89 (24%)	34 (24%)	273 (17%)	
> 30 rpm	240 (66%)	96 (68%)	1282 (79%)	
PEEP^j^				
5–9 cmH_2_O	151 (40%)	61 (41%)	547 (34%)	0.089
10–13 cmH_2_O	174 (46)	62 (42%)	828 (51%)	
14–17 cmH_2_O	53 (16,7%)	24 (16%)	240 (15%)	
> 18 cmH_2_O	1 (0,3%)	1 (1%)	9 (0,6%)	
FIO_2_^k^				
≤ 30%	39 (10%)	16 (11%)	83 (5%)	< 0.001^a,c^
31%–59%	210 (56%)	91 (61%)	626 (39%)	
60%–79%	99 (26%)	22 (15%)	439 (27%)	
≥ 80%	30 (8%)	19 (13%)	478 (29%)	
PaO_2_/FiO_2_^l^	186 [130–251]	175 [132–241]	147 [100–207]	< 0,001^a,c^
Prone MV Y/N (%)^m^	87 (23%)/293 (77%)	43 (29%)/105 (71%)	319 (19%)/1351 (81%)	0.007^c^
Tracheostomy Y/N (%)^n^	120 (32%)/260 (68%)	101 (69%)/46 (31%)	148 (9%)/1521 (91%)	< 0.001^a,b,c^
Time to TQT	16 [13–19]	18 [15–22]	16 [14–19]	0.001^b,c^
Ventilatory mechanics				
Cest^o^				
≤ 30 cmH_2_O	81 (39%)	37 (46%)	316 (38%)	0.288
33–49 cmH_2_O	113 (54%)	40 (50%)	459 (55%)	
≥ 50 cmH_2_O	14 (7%)	3 (4%)	55 (7%)	
Driving pressure^p^				
≤ 10 cmH_2_O	73 (33%)	26 (29%)	304 (34%)	0.465
11–15 cmH_2_O	133 (61%)	52 (59%)	486 (54%)	
> 15 cmH_2_O	14 (6%)	11 (12%)	112 (12%)	
Hospitalization data				
Period of infection				
1st^b^ wave	59 (16%)	23 (15%)	247 (15%)	0.682
2nd^b^ wave	297 (78%)	111 (75%)	1292 (77%)	
3rd^b^ wave	24 (6%)	14 (9%)	131 (8%)	
Time to OTI (days)	10 [7–13]	9 [6–13]	9 [5–14]	0.302
IMV time (days)	12 [7–20]	25 [14–37]	7 [3–13]	< 0.001^a,b,c^
ICU time (days)	16 [10–27]	29 [14–45]	5 [0–12]	< 0.001^a,b,c^
Length of stay (days)	24 [16–37]	34 [22–55]	9 [5–16]	< 0.001^a,b,c^
Outcome				
Discharge	319 (84%)	56 (38%)	0 (0%)	< 0.001^a,b,c^
Death	58 (15%)	92 (62%)	1581 (95%)	
Transfer	3 (1%)	0 (0%)	89 (5%)	
Rehospitalization Y/N (%)	8 (2%)/372 (98%)	3 (2%)/145 (98%)	13 (1%)/1657 (99%)	0.042^a^

*Note:* Cest, static compliance.

Abbreviations: BMI, body mass index; CT, computed tomography; F, female; FiO_2_, fraction of inspired oxygen; FW, failed ventilatory weaning; ICU, intensive care unit; IMV, invasive mechanical ventilation; kg, kilograms; M, male; m^2^, square meter; NCW, no criteria for ventilatory weaning; NIMV, noninvasive mechanical ventilation; OTI, orotracheal intubation; PaO_2_, partial pressure of oxygen; PCV, pressure‐controlled ventilation; PEEP, positive end‐expiratory pressure; PSV, pressure support ventilation; SpO_2_, peripheral oxygen saturation; SW, successful ventilatory weaning; VCV, volume‐controlled ventilation.

^a^Difference between SW and NCW.

^b^Difference between SW and FW.

^c^Difference between FW and NCW.

^d^Missing data (*n* = 236).

^e^Missing data (*n* = 59).

^f^Missing data (*n* = 388).

^g^Missing data (*n* = 46).

^h^Missing data (*n* = 155).

^i^Missing data (*n* = 74).

^j^Missing data (*n* = 47).

^k^Missing data (*n* = 46).

^l^Missing data (*n* = 79).

^m^Missing data (*n* = 79).

^n^Missing data (*n* = 2).

^o^Missing data (*n* = 1080).

^p^Missing data (*n* = 987).

The likelihood of failing weaning was higher among individuals with DP > 15 cmH_2_O. Additionally, the probability of WF increased by 2.8% for each year of age and by 10.3% for each additional day of IMV.

**Figure 2:**
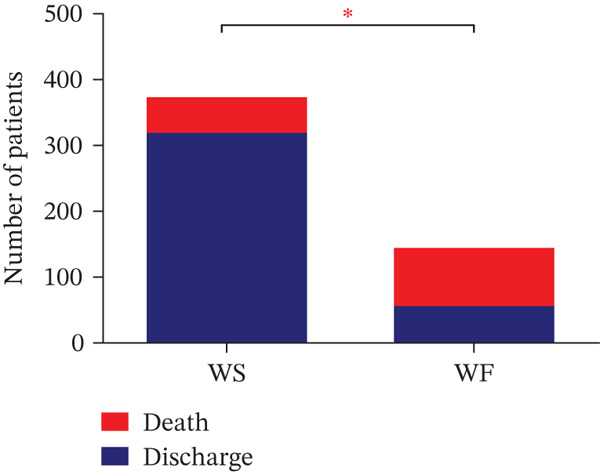
Difference in the proportion of individuals who died or were discharged from the hospital between the WS and WF groups.  ^∗^WS, weaning success; WF, weaning failure.

There was a higher likelihood of WF among individuals who spent less time in the hospital, and the proportion of individuals who progressed to death was significantly higher in this group (WF: 62% vs. WS: 38%; *p* < 0.0001) (Figure 2).

### 3.1. Kaplan–Meier Survival Analysis Between Individuals With Successful and Failed Weaning

**Figure 3:**
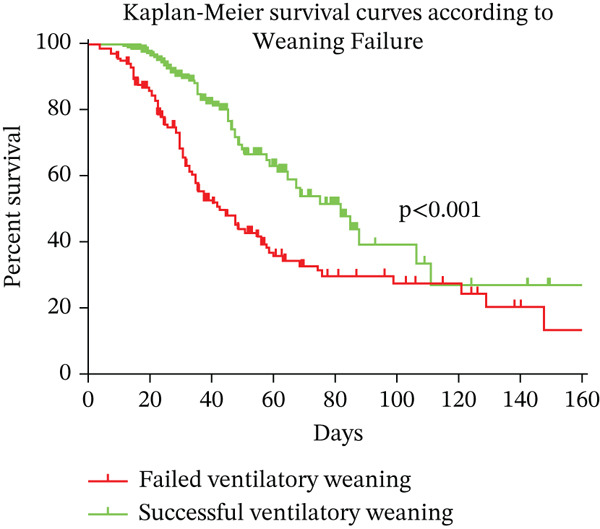
Kaplan–Meier survival curves for mortality stratified by ventilatory weaning.

The results of the Kaplan–Meier survival curves for mortality are presented, comparing WS and WF in Figure 3. The curves indicate a reduced survival time in subjects with WF (74 days; 95% CI: 58–86) compared with those with WS (114 days; 95% CI: 84–146) (*p* < 0.001).

### 3.2. Association With Weaning Variables

In the univariate analysis, several variables were significantly associated with WF in patients with COVID‐19 (Table 2). Older age (OR: 1.028; 95% CI: 1.014–1.042; *p* < 0.05), functional impairment (OR: 2.802; 95% CI: 1.029–7.629; *p* < 0.05), longer hospital stay (OR: 1.016; 95% CI: 1.009–1.024; *p* < 0.05), and prolonged mechanical ventilation duration (OR: 1.054; 95% CI: 1.040–1.069; *p* < 0.05) were associated with higher odds of WF. Additionally, patients who received a tidal volume > 8 mL/kg of predicted body weight had increased odds of WF (OR: 2.464; 95% CI: 1.062–5.721; *p* < 0.05). In the multivariate model, age remained an independent predictor of WF (OR: 1.028; 95% CI: 1.005–1.052; *p* < 0.05). Interestingly, a longer hospital stay showed a protective effect (OR: 0.963; 95% CI: 0.937–0.990; *p* < 0.05), whereas mechanical ventilation time continued to be positively associated with failure (OR: 1.103; 95% CI: 1.059–1.149; *p* < 0.05). Driving pressure > 15 cmH_2_O also emerged as a significant independent predictor (OR: 3.750; 95% CI: 1.344–10.466; *p* < 0.05).

**Table 2:** Predictors of weaning failure in COVID‐19 patients.

	Univariate	Multivariate
	OR [IC 95%]	OR [IC 95%]
Age (years)	1.028 [1.014–1.042] ^∗^	1.028 [1.005–1.052] ^∗^
Gender (F/M)	1.456 [0.989–2.144]	
BMI (kg/m^2^)	0.974 [0.944–1.004]	
Comorbidities (*n*)	1.059 [0.917–1.223]	
Functionality (I/PI/D)^a^	2.802 [1.029–7.629] ^∗^	
Length of stay (days)	1.016 [1.009–1.024] ^∗^	0.963 [0.937–0.990] ^∗^
Time to OTI (days)	0.973 [0.939–1.007]	
IMV time (days)	1.054 [1.040–1.069] ^∗^	1.103 [1.059–1.149] ^∗^
Tidal volume (mL)^b^	2.464 [1.062–5.721] ^∗^	
Driving pressure (cmH_2_O)^c^	2.206 [0.890–5.468]	3.750 [1.344–10.466] ^∗^

Abbreviations: BMI, body mass index; COVID‐19, coronavirus disease‐19; D, dependent; F, female; I, independent; IMV, invasive mechanical ventilation; M, male; OTI, orotracheal intubation; PI, partially independent.

^a^Belonging to the dependent category.

^b^Belonging to the category that received tidal volume > 8 mL/kg of predicted weight.

^c^Belonging to the category with driving pressure > 15 cmH_2_O.

^∗^ Univariate model, *p* < 0.15. Multivariable model, *p* < 0.05. Variables with *p* < 0.15 in the univariate analysis were included in the multivariable model, with statistical significance set at *p* < 0.05.

## 4. Discussion

This study is aimed at identifying the predictors of WF from IMV in patients with COVID‐19 and their association with mortality. Advanced age, the length of hospitalization, prolonged IMV duration, and compromised respiratory mechanics were the predictors of WF. Furthermore, individuals who experienced WF were typically older and male, exhibited greater comorbidity burden, had lower functional status, and presented a higher mortality rate compared with those who successfully weaned.

Our results showed that age is a reliable and independent predictor of WF in patients on IMV, which could increase the death rates. The association between advanced age and various clinical outcomes during hospitalization has been extensively studied, with a primary focus on length of stay and mortality [30]. The risk of ventilatory failure increases with each decade after 60 years old due to a range of factors associated with the aging process, including a decline in respiratory muscle strength, sarcopenia, frailty, thoracic stiffness, decreased lung compliance, and the presence of chronic diseases [33, 34].

Previous studies have shown that longer hospital stays negatively impact the weaning process, particularly in individuals with COVID‐19 [34]. Kolck et al. reported an exponential increase in the risk of WF and mortality among patients with prolonged hospitalization [36, 37]. Interestingly, our results demonstrated a higher likelihood of WF among individuals who spent less time in the hospital. This finding may be explained by the increased mortality rate and consequent shorter duration of hospitalization among those patients who failed weaning.

It is well known that extended hospital stays are associated with a higher chance of remaining on mechanical ventilation for a long time because they increase the likelihood of complications such as infections, ICU‐acquired weakness, and adverse events [37]. On the other hand, in addition to the length of hospital stay, the duration of IMV emerged as one of the most influential predictors of WF.

IMV duration was independently associated with WF. However, this variable likely reflects downstream consequences of severe respiratory illness, complications, or early difficulty in the weaning process, rather than a true preexisting determinant of weaning outcome. Patients who are more severely ill typically require longer ventilatory support and are also inherently more prone to fail weaning. Therefore, IMV duration should be interpreted as a marker of underlying severity and illness trajectory rather than a causal predictor.

Regarding the association between hospital length of stay and WF, the univariate analysis suggested that longer hospitalization was associated with a higher likelihood of WF. However, in the multivariate analysis, this association reversed, and length of stay was independently associated with lower odds of WF. This shift likely reflects confounding and collinearity [38, 39] with markers of disease severity. In the unadjusted analysis, sicker patients tend both to remain hospitalized longer and to be more prone to WF, creating a positive crude association. Once severity and related factors are accounted for in the multivariate model, the independent effect of length of stay becomes clearer, suggesting that additional hospital days may reflect clinical stability, opportunities for recovery, or progression through rehabilitation, thereby reducing the likelihood of WF. Each additional day on mechanical ventilation was an independent factor for WF from mechanical ventilation, rising by over 10%. Similar results were observed in the literature, confirming that prolonged ventilatory support makes weaning more difficult or not possible, as is the case for most of the population included in our study.

Amir et al. observed that those individuals who were ventilated for longer than 2 weeks had higher rates of WF [40], even after being adjusted for age, comorbidities, and the severity of ARDS. Another study observed that more than 10 days in mechanical ventilation increased the risk of WF by around 50% [41]. Additionally, the risk of WF rises significantly with each extra day spent on IMV; prolonged ventilation may cause ventilator‐induced diaphragm dysfunction, which reduces respiratory strength and thoracic compliance, potentially making weaning impracticable [20, 40, 42].

In our study, driving pressure emerged as the variable most strongly associated with WF among patients with COVID‐19. Multivariate regression analysis confirmed that higher DP remained independently associated with WF after adjusting for other confounders. Consistent with previous reports [43, 44] patients with DP above 15 cmH_2_O, reflecting reduced lung compliance and increased alveolar stress, were more likely to fail weaning. These results highlight DP as an important marker in the weaning process; however, it should be viewed primarily as an indicator of underlying disease severity rather than a direct causal factor. Elevated DP reflects impaired respiratory mechanics, and patients with greater pulmonary compromise are naturally at higher risk of WF. We observed that those patients with COVID‐19 who experienced unsuccessful weaning had a higher DP than those who experienced successful weaning. These findings support the idea that DP plays a crucial role in the weaning process. In this line, Yan et al. found that high DP was one of the primary predictors of WF, after adjusting for different confounders [45]. Another study showed that DP variation in the first 4 days was an important predictor of WF [46].

We found that patients who failed weaning had a higher rate of hospital readmission and mortality, suggesting that their clinical vulnerability persisted even after they were discharged. Previous studies reported that patients with COVID‐19 who received mechanical ventilation and higher IMV duration had a threefold increased risk of readmission compared with those who did not receive ventilation [47, 48]. On the other hand, mortality among patients with COVID‐19 who failed weaning from IMV was elevated, reaching over 60%, significantly higher than the 15% observed in the successfully weaned group.

A current study reported that among patients requiring prolonged mechanical ventilation, over 45% died either during their ICU stay or within 6 months, especially those unable to be weaned [49]. When compared with the high mortality rates observed in our cohort, these findings reinforce that WF is intrinsically linked to poor prognosis, with a heightened risk of complications such as ICU‐acquired muscle weakness, ventilator‐induced lung injury infections, and other conditions. Moreover, patient‐centered care strategies such as structured postdischarge follow‐up and transfer to specialized respiratory rehabilitation units may be essential to reduce the elevated mortality burden in this population [32, 40].

Finally, despite concerted efforts, this study has certain limitations, particularly the heterogeneity of the sample and the inclusion of a substantial number of patients who did not meet established weaning criteria. This limited the possibility of more detailed analyses and may reduce the generalizability of the results to the broader population treated at this institution. Nevertheless, the findings offer clinically relevant insights into the management of critically ill COVID‐19 patients undergoing IMV.

Early identification of predictors of WF may help healthcare teams recognize high‐risk individuals and tailor interventions accordingly [28]. Future studies should investigate personalized weaning protocols in patients with COVID‐19 or other causes of ARDS, focusing on factors such as baseline functional status, CT severity scores, and the evolution of compliance and driving pressure over time. Additionally, longitudinal studies are needed to evaluate the impact of structured post‐ICU rehabilitation programs on reducing mortality and hospital readmissions in this high‐risk population. Refining weaning criteria and optimizing supportive care could ultimately improve not only survival rates but also long‐term functional outcomes.

## 5. Conclusion

In individuals with COVID‐19, the predictors of WF were advanced age, the length of hospitalization, prolonged IMV duration, and compromised respiratory mechanics. Furthermore, individuals who experienced WF were typically older and male, exhibited greater comorbidity burden, had lower functional status, and presented a higher mortality rate compared with those who successfully weaned.

**Nomenclature**
ARDS: acute respiratory distress syndrome
BMI: body mass index
CT: computed tomography
DP: driving pressure
FiO_2_
: fraction of inspired oxygen
HFNC: high‐flow nasal cannula
ICU: intensive care unit
IMV: invasive mechanical ventilation
NCW: no criteria for weaning
NIV: noninvasive ventilation
OR: odds ratio
PaO_2_
: partial pressure of oxygen
PEEP: positive end‐expiratory pressure
PSV: pressure support ventilation
RR: respiratory rate
SARS‐CoV‐2: Severe Acute Respiratory Syndrome Coronavirus 2
SBT: spontaneous breathing trial
WF: weaning failure
WS: weaning success
VAP: ventilator‐associated pneumonia
VCV: volume‐controlled ventilation

## Funding

This study was financed in part by the Coordination for the Improvement of Higher Education Personnel–Brazil (CAPES) (Finance Code 001), Superintendência de Ciência, Tecnologia e Ensino Superior (SETI), Fundação Araucária and Universidade Estadual de Londrina (PROPPG) as partial support.

## Conflicts of Interest

The authors declare no conflicts of interest.

## Data Availability

The data that support the findings of this study are available on request from the corresponding author. The data are not publicly available due to privacy or ethical restrictions.
